# Different dietary methionine to lysine ratios in the lactation diet: effects on the performance of sows and their offspring and methionine metabolism in lactating sows

**DOI:** 10.1186/s40104-019-0373-2

**Published:** 2019-08-22

**Authors:** Hongkui Wei, Xichen Zhao, Mao Xia, Chengquan Tan, Jun Gao, John K. Htoo, Chuanhui Xu, Jian Peng

**Affiliations:** 1 Department of Animal Nutrition and Feed Science, College of Animal Science and Technology, Huazhong Agricultural University, Wuhan, People’s Republic of China; 2 Department of Animal Nutrition and Feed Science, College of Animal Science, South China Agricultural University, Guangzhou, People’s Republic of China; 3 Evonik Degussa (China) Co., Ltd, Beijing, People’s Republic of China; 4 Evonik Nutrition & Care GmbH, Hanau-Wolfgang, Essen, Germany; 5 The Cooperative Innovation Center for Sustainable Pig Production, Wuhan, People’s Republic of China

**Keywords:** Lactation, Methionine, Met to Lys ratios, Oxidative stress, Piglet growth performance, Sow performance

## Abstract

**Background:**

Over the last decade, the nutritional requirements of lactating modern genotype sows have increased. The current nutritional recommendations might be unable to meet the needs of increased litter size and milk production, and thus the nutritional requirements need to be re-evaluated. The current study was conducted to investigate the effects of dietary methionine to lysine (Met:Lys) ratios on the performance of and methionine metabolism in lactating sows.

**Results:**

During the 1^st^ week of lactation, piglets reared on sows in the 0.37 to 0.57 Met:Lys ratio groups grew faster than those reared on sows in the control group (0.27) (*P* < 0.01). The 0.37-ratio group showed increased levels of GSH-Px in plasma during lactation (*P* < 0.01) and decreased concentrations of urea nitrogen in the plasma of sows (*P* < 0.05). Compared with the 0.27-ratio group, the levels of T-AOC and GSH-Px in the plasma and homocysteine in the milk of lactating sows were significantly increased in sows in the 0.47-ratio group (*P* < 0.01). In sows fed a 0.57-ratio diet, the levels of glutathione and taurine in the plasma and milk were improved significantly during lactation. However, the content of TBARS in the blood (*P* < 0.05 at day 7 and *P* = 0.06 at weaning day) was increased (*P* < 0.01). Moreover, there were linear increases in the levels of homocysteine in the blood and milk of sows during the lactation period (*P* < 0.01) with increased dietary Met:Lys ratios in the lactation diet.

**Conclusions:**

The current study indicated that increasing the dietary Met:Lys ratio (0.37~0.57) in the lactation diet had no significant effect on the overall performance of sows or the colostrum and milk composition, but it increased piglet mean BW and piglet ADG during the first week of lactation. Increasing dietary methionine levels had no significant effect on antioxidant function in lactation sows, even though it increased levels of GSH and GSH-Px in the plasma of sows during lactation. However, the content of homocysteine in the plasma and milk increased during lactation due to a high level of dietary methionine.

## Introduction

During the last decade, litter size and milk production of modern genotype sows have increased drastically, and thus, the nutritional requirements may need to be re-evaluated [1]. Methionine, a sulfur amino acid (SAA), plays an important role in human and animal nutrition and health [2, 3]. Adequate amounts of methionine are necessary for protein synthesis [4, 5]. Previous studies that analyzed the profiles of amino acids in sow milk or other tissues have suggested that the supply of methionine in the diet may not match the requirements of sows. The Met:Lys ratio calculated from physiological IAA needs for milk synthesis by lactating sows predicted that the maximal mammary uptake of plasma IAA is 0.34 [6], which is higher than that recommended by the NRC (2012). Hence, the dietary Met:Lys ratio recommended by the NRC (2012) may be insufficient to achieve optimal lactation performance by hyperprolific sows.

Notably, systemic oxidative stress increases during lactation, and antioxidant nutrients, such as vitamin A, in circulation are substantially reduced and do not fully recover until weaning [7, 8]. Therefore, lactating sows may need more antioxidants to relieve oxidative stress. It has been demonstrated that methionine contributes to antioxidant function by forming cysteine or glutathione via sulfur metabolism [2]. However, homocysteine, an intermediate metabolite in methionine metabolism, potentially contributes to the formation of oxygen free radicals and peroxynitrite as well as increased oxidative stress by inhibiting the expression of antioxidant enzymes [9], thus leading to endothelial cytotoxicity [10, 11]. Therefore, the effect of dietary methionine levels on oxidant status of lactating sows might need to evaluation.

Consequently, the current study aimed to investigate the effects of increased dietary Met:Lys ratios in lactation diets on the performance and methionine metabolism of sows. It was hypothesized that providing a higher and suitable dose of methionine than that recommended by the NRC (2012) in the diet of lactating sows is needed to meet the requirements for lactation, improve the metabolic status of sows and increase sow productivity. Moreover, it may also help to alleviate lactation oxidative stress and improve sow reproductive performance.

## Methods

### Experimental design, animals, and housing

A total of 130 multiparous sows (Large White) with a parity of 3 to 6 (4.22 ± 0.81) were randomly allocated to 4 dietary treatments (*n* = 33, 32, 32, 33) with 4 dietary SID Met:Lys ratios (0.27, 0.37, 0.47 and 0.57:1) based on body weight. The sows in the trial were moved to the farrowing unit 4 days before expected parturition and kept in individual farrowing crates with stalls (2.2 m × 0.7 m) in pens that provided space on both sides of the stall (2.2 m × 0.5 m) for the piglets after birth. The animals were studied throughout lactation after the determination of backfat thickness (measured from the back line 6.5 cm, P2, RENCO LEAN-METER Digital Backfat Indicator S/N 61323) and the body surface of all sows (particularly the claws and the teats) were washed and disinfected with peracetic acid before farrowing. The temperature in the farrowing unit was controlled at 15–22 °C, and each pen had a covered area in the corner equipped with a heating lamp for the piglets. Twenty-four hours postpartum (day 2 after birth), the litters of the experimental sows were standardized to at least 11 piglets by cross-fostering within the group. All piglets were given iron injections and were tail docked, and males were surgically castrated between days 5 and 7 postpartum. All animals were managed according to the general routines of the herd. The piglets were weaned on day 21 after farrowing.

### Feeding system and diets

From 1 day before expected parturition to the end of the experiment, the sows were fed the corresponding treatment diets. The diets were formulated using standard ileal digestibility values of individual ingredients to be isoenergetic and isonitrogenous (Table 1). From farrowing to day 3 postpartum, the daily feed allowance was 3, 3.5, 4, and 4.5 kg/d on day 0, 1, 2, and 3, respectively. From day 4 until weaning, the sows had *ad libitum* access to feed and water. All sows were fed 3 times a day (07:00, 14:00, 21:00). Creep feed was not offered to litters during lactation.

**Table 1:** Ingredients and nutrient composition of diets

Items	Met:Lys			
	0.27	0.37	0.47	0.57
Ingredients, %				
Corn	42.75	42.74	42.85	42.94
Soybean meal (46% CP)	24.3	24.2	24	23.9
Red sorghum	13.4	13.4	13.4	13.4
Shelly barley	12.7	12.7	12.7	12.7
Soybean oil	2.2	2.2	2.2	2.1
Limestone	1.67	1.68	1.68	1.68
MDCP	1.45	1.45	1.45	1.46
Premix^a^	1	1	1	1
Salt	0.3	0.3	0.3	0.3
Methionine	–	0.09	0.18	0.27
Lysine (98%)	0.15	0.16	0.16	0.17
Mold Inhibitor	0.08	0.08	0.08	0.08
Total	100	100	100	100
Composition (analysis)				
Lys, %	1.05	1.03	1.02	1.09
Met, %	0.24	0.31	0.39	0.55
Cys, %	0.22	0.22	0.21	0.27
SAAs, %	0.46	0.53	0.60	0.82
Met/Lys	0.23	0.30	0.38	0.50
Thr, %	0.65	0.65	0.65	0.68
Trp, %	0.19	0.18	0.19	0.20
Val, %	0.77	0.77	0.77	0.79
Ile, %	0.68	0.67	0.67	0.68
Leu, %	1.53	1.50	1.49	1.53
Arg, %	1.00	1.01	1.00	1.02
His, %	0.46	0.46	0.45	0.46
Composition (calculated)^b^				
NE, kcal/kg	2451	2452	2455	2452
CP, %	17.3	17.32	17.28	17.3
Ca, %	0.99	0.99	0.99	0.99
Available phosphorus, %	0.44	0.44	0.44	0.44
SID Lys, %	0.88	0.88	0.88	0.88
SID Met, %	0.24	0.32	0.41	0.5
SID Cys, %	0.25	0.25	0.24	0.24
SID SAAs, %	0.48	0.57	0.66	0.74
SID Met/Lys	0.27	0.36	0.47	0.57
SID Thr, %	0.54	0.54	0.54	0.54
SID Trp, %	0.18	0.18	0.18	0.18
SID Val, %	0.70	0.70	0.69	0.69
SID Ile, %	0.63	0.62	0.62	0.62
SID Leu, %	1.32	1.32	1.31	1.31
SID BCAA, %	2.65	2.64	2.63	2.62
SID Arg, %	0.99	0.99	0.99	0.98
SID His, %	0.40	0.39	0.39	0.39

^a^Provided per kilogram of the diet: Cu 30 mg; Fe 160 mg; Zn 150 mg; Mn 50 mg; I 0.53 mg; Se 0.53 mg; Co 0.75 mg; Cr 0.22 mg; vitamin A 1.271 × 10^4^U; vitamin D_3_ 2853 U; vitamin E 180 mg; vitamin K_3_ 3.85 mg; vitamin B_1_ 1.6 mg; vitamin B_2_ 5.75 mg; vitamin B_6_ 2.88 mg; vitamin B_12_ 0.02 mg; nicotinamide 32 mg; pantothenic acid 20 mg; folic acid 3.2 mg; biotin 0.44 mg; vitamin C 450 mg; choline 1800 mg

^b^Calculated chemical concentrations using values for feed ingredients from the NRC (2012)

### Recording and sampling

The body weight of all sows was measured at day 110 of gestation and at weaning. The backfat thickness of all sows was measured at day 110 of gestation and the day of farrowing and weaning. Piglet weights were recorded separately at farrowing, day 7 of lactation, day 14 of lactation and weaning. The feed intake of sows was recorded every day, and the weaning to estrus interval was also recorded. At farrowing, day 7 and weaning day, blood samples were collected from each sow 2 h after the morning feeding using a 10-mL syringe and 5-mL vacuum blood collection tube containing an anticoagulant (EDTAK_2_) and then placed on ice immediately. At least 10 sows with similar parity per diet group were selected randomly at each sampling time point. Plasma samples were obtained by centrifugation (HC-2066, China) at 1500×*g* for 15 min at room temperature, aliquoted into 0.5-mL microcentrifuge tubes, and then frozen at − 20 °C until analysis. The plasma was analyzed for the contents of plasma urea nitrogen, malondialdehyde, 8-hydroxy-deoxyguanosine, glutathione peroxidase, reactive oxygen species, total antioxidative capacity, and methionine metabolites, such as homocysteine and taurine. Before sample collection, 10 U oxytocin was injected in the sow ear vein, and colostrum and milk samples (25~30 mL) were collected from the anterior, middle, and posterior teats from one side of the sow using a 50-mL centrifuge tube; at least 10 sows with similar parity per diet group were selected randomly at each sampling time-point. The well-mixed samples were collected and then immediately frozen at − 20 °C until analysis.

### Chemical analyses

The milk composition was determined by near-infrared reflectance spectroscopy with a Foss Milkoscan FT+ (CombiFT+ 200, Denmark). Before analysis, 5 mL thawed fresh milk per sample was aliquoted into a 50-mL centrifuge tube (sterilized), and 20 mL distilled water was added to dilute the sample. However, before the analysis of homocysteine, glutathione, methionine, cysteine, and taurine in milk, the whey was collected and centrifuged at 1500×*g* at 4 °C for 15 min (5804R, Eppendorf, Germany). Homocysteine and glutathione concentrations in the milk and plasma were determined using a homocysteine detection kit (E031) and a reduced glutathione assay kit (A006–2) (Jiancheng Bioengineering Limited, China), respectively, according to the manufacturer’s instructions. The assay for homocysteine was based on the method of enzymatic cycling, and the assay for glutathione was based on the microenzyme label method. The concentrations of methionine, cysteine and taurine in plasma and milk were determined by ion-exchange chromatography using an L-8800 high-speed amino acid analyzer (Hitachi, Japan), as described previously [12]. The plasma levels of GSH-Px (A005), T-AOC (A015) and TBARS (A003–1) were determined using a detection kit (Jiancheng Bioengineering Limited, China) according to the manufacturer’s instructions. Plasma contents of 8-OHdG (Dobio Biotech Co., Ltd., Shanghai, China) and ROS (MDL Biotech Co., Ltd., Beijing, China) were determined by the pig ELISA kit strictly in accordance with the manufacturer’s instructions. All samples were analyzed in duplicate, and all frozen plasma and whey samples were thawed at 4 °C, mixed thoroughly and centrifuged at 10,000 r/min (5418, Eppendorf, Germany) for 10 min before the assay.

### Calculations and statistical analyses

All calculations and statistical analyses were performed using SAS software (SAS 8.0, Inst, Inc., Cary, NC) with the individual sow as the experimental unit. Mixed procedures and ANOVA were also used in the analyses.

Piglet growth performance was analyzed using the following model:

$$ {Y}_{\mathrm{i}\mathrm{j}}=\mu +{\alpha}_{\mathrm{i}}+{\beta}_{\mathrm{i}\mathrm{j}}+{X_{\mathrm{i}\mathrm{j}}}^{(1)}+{X_{\mathrm{i}\mathrm{j}}}^{(2)} $$

where *Y*_ij_ is the response variable, *μ* is the overall mean, *α*_i_ is the fixed effect of diet, *β*_ij_ is the random effect of parity, *X*_ij_^(1)^ is the covariate that represents the weight or litter weight of piglets after cross-fostering, and *X*_ij_^(2)^ is the covariate that represents the number of piglets.

Milk composition was analyzed using the following model:

$$ {Y}_{\mathrm{i}\mathrm{j}}=\mu +{\alpha}_{\mathrm{i}}+{X_{\mathrm{i}\mathrm{j}}}^{(1)}+{X_{\mathrm{i}\mathrm{j}}}^{(2)} $$

where *Y*_ij_ is the response variable, *μ* is the overall mean, *α*_i_ is the fixed effect of diet, *X*_ij_^(1)^ is the covariate that represents the parity of the sow, and *X*_ij_^(2)^ is the covariate that represents the BW of the sow.

Data obtained by the mixed procedure are shown as Lsmeans and SEM, and those obtained by ANOVA are shown as the mean and SEM. Statistical significance was declared at *P* < 0.05, and tendencies were declared at 0.05 < *P <* 0.10. Multiple comparisons were made when the ANOVA indicated significant differences. Tukey’s test was used in multiple comparisons of means to adjust the *P*-values when using a mixed model procedure for data analysis. Duncan’s test was used for One-way ANOVA.

## Results

### Sow performance

The body weight of the sows in different groups did not differ significantly at day 110 of lactation, day of parturition and weaning day, and weight loss during lactation did not differ (Table 2). Moreover, the dietary Met:Lys ratio did not affect backfat thickness, WEI or total milk yield during lactation (Table 2).

**Table 2:** Effect of Met:Lys ratio in the lactation diet on the performance of sows

Items	Met:Lys				SEM	*P*-value		
	0.27	0.37	0.47	0.57		Linear	Quadratic	Feed
Basic information								
No. of sows	33	32	32	33				
Culled^a^ during lactation	4	1	–	1				
Average body weight, kg	266.70	266.50	266.70	266.40	1.64			
Average parity	4.40	4.10	4.00	4.38	0.07			
BW of sows, kg								
Day 110 of lactation	268.9	266.4	265.8	266.8	1.7	0.70	0.80	0.93
Parturition	264.8	262.3	261.8	262.8	1.7	0.70	0.81	0.93
Weaning	245.2	243.9	244.5	247.3	1.8	0.70	0.77	0.92
Loss during lactation	−19.6	−18.4	−17.2	−15.4	1.4	0.30	0.57	0.77
WEI of sows, d								
No. of sows	29	31	31	32				
Within 1 week	4	5	4	5	0.13	0.97	0.99	0.93
After 1 week	6	8	5	6	0.51	0.80	0.73	0.22
Estrus rate of sows, %^b^								
Within 1 week	89.66	77.42	93.55	90.63		0.66	0.86	0.22
After 1 week	96.55	100.00	100.00	100.00		0.20	0.26	0.35
Sow backfat thickness, mm								
No. of sows	32	32	32	33				
Day 110 of lactation	15.72	15.97	15.72	15.33	0.24	0.50	0.65	0.83
Parturition	14.94	15.38	15.19	14.76	0.27	0.80	0.69	0.86
No. of sows	29	31	32	33				
Weaning	14.41	15.29	14.94	14.42	0.26	0.90	0.40	0.57
Loss during lactation	−0.59	−0.19	−0.25	−0.33	0.21	0.70	0.81	0.92
Lactation performance, kg								
Total milk yield^c^	203.59	207.18	213.29	210.94	3.26	0.56	0.80	0.70
Average daily milk yield	11.10	11.34	11.62	11.44	0.08	0.59	0.85	0.97

*SEM* standard error of the mean, *BW* body weight, *WEI* weaning to estrous interval; Annotation: significance test for estrus rate of each group derived from the chi-square test; N indicates the number of sows

^a^The sows eliminated in each group for death, lameness, limb hoof disease and so forth during lactation

^b^Significance test for estrus rate of each group derived from the chi-square test

^c^The result of total milk yield during the whole lactation period is shown as Lsmeans and derived from the mixed procedure of SAS (8.0). The formula for the calculation of total milk yield is total milk yield (kg) = piglet ADG × litter size × lactating days × 4 [13]

### Piglet performance

The effects of dietary Met:Lys ratios in the lactation diet on piglet growth performance during lactation are shown in Table 3. There were no differences in piglet numbers, number of piglets weaned or piglet weights at day 14 after birth. However, the piglets reared on sows in the 0.37- or 0.57-ratio groups grew faster than those reared on sows in the control group (0.27) during the 1^st^ week of lactation (*P* < 0.01). Only the piglets in the 0.47-ratio group had a significantly higher weaning weight than those in the 0.27-ratio group. However, the average daily gain (ADG) of piglets and weaned litter weights were unaffected by dietary treatments (*P* > 0.05).

**Table 3:** Effect of dietary Met:Lys ratio in the lactation diet on growth performance of piglets

Items	Met:Lys				SEM	*P*-value		
	0.27	0.37	0.47	0.57		Linear	Quadratic	Feed
No. of sows	27	30	28	31				
Litter size, number/litter								
After cross-fostering	11.26	11.5	11.18	11.42	0.09	0.85	0.98	0.58
At day 7	10.63	10.87	10.14	10.45	0.12	0.24	0.49	0.15
At day 14	10.22	10.47	9.68	10.13	0.12	0.34	0.56	0.11
Pigs weaned	9.89	10.13	9.36	9.97	0.13	0.68	0.68	0.15
Survival rate of piglets, %	87.91	88.20	84.29	87.45	1.02	0.62	0.65	0.53
Piglet mean BW, kg^e^								
After cross-fostering	1.78	1.78	1.74	1.76	0.01	0.24	0.41	0.60
At day 7^c^	2.78^B^	3.13^A^	3.01^AB^	3.04^A^	0.02	0.15	0.33	< 0.0001
At day 14	4.78	5.11	5.07	5.07	0.03	0.26	0.49	0.12
At day 21^d^	6.85^b^	7.07^ab^	7.55^a^	7.25^ab^	0.04	0.06	0.08	0.02
Piglet ADG, g/d^e^								
Week 1^c^	143^B^	191^A^	174^AB^	179^A^	2	< 0.01	< 0.001	< 0.001
Week 2	280	288	289	285	2	0.83	0.98	0.94
Week 3	289	300	294	307	2	< 0.001	< 0.001	0.60
Days 1–21	243	261	256	260	1	0.01	< 0.01	0.25
Litter weight, kg^e^								
After cross-fostering	20.48	20.34	19.46	20.04	0.33	0.47	0.66	0.71
At day 7	31.54	32.13	31.25	31.54	0.52	0.47	0.75	0.88
At day 14	51.6	51.63	50.11	50.86	0.76	0.38	0.60	0.84
At day 21	70.53	71.24	69.10	71.23	1.02	0.80	0.84	0.85

*SEM* standard error of the mean, *BW* body weight, *ADG* average daily gain

^c, d^Means in the same row with different letters differ significantly; the different lowercase letters represent *P* < 0.05, and the different capital letters represent *P* < 0.01

^e^The results of piglet mean BW, ADG and litter weight are expressed as Lsmeans

### Colostrum and milk composition and PUN

**Fig. 1:**
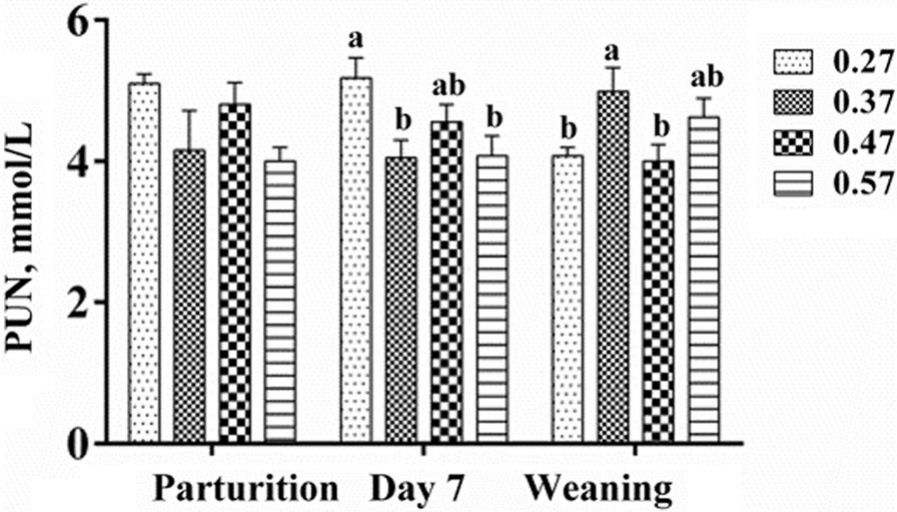
Dietary effect on the concentration of urea nitrogen in plasma. Blood samples were collected from the ear vein of sows at the morning feeding and 2 h later. Data are presented as the means±SEM, *n* ≥ 10/lactation diet. ^a-b^Means in the same row with different letters differ significantly, *P* < 0.05

No difference was observed in the level of lactoprotein in the colostrum or milk. Similarly, fat, lactose, non-fat solids and total solids in the colostrum or milk were not influenced by treatments (Table 4). As shown in Fig. 1, the levels of urea nitrogen in the plasma of sows in the 0.37- and 0.57-ratio groups were lower than those in the 0.27-ratio group at day 7. However, on the day of weaning, only the sows fed with the 0.37-ratio diet had a lower content of plasma urea nitrogen than those fed the 0.27-ratio diet (*P* < 0.05).

**Table 4:** Influence of dietary Met:Lys ratio in lactation diet on composition of colostrum and milk

Items		Met:Lys				SEM	*P*-value		
		0.27	0.37	0.47	0.57		Linear	Quadratic	Feed
No. of sows^a^									
Fat, %	Colostrum	5.57	6.05	5.49	5.22	0.28	0.47	0.74	0.82
	Milk of day 7	7.02	7.86	7.48	7.98	0.23	0.20	0.41	0.45
	Milk of day 20	6.94	6.93	7.45	7.27	0.14	0.23	0.47	0.46
Protein, %	Colostrum	12.07	13.10	11.23	13.70	0.52	0.46	0.63	0.33
	Milk of day 7	6.31	7.09	6.49	6.96	0.14	0.27	0.51	0.2
	Milk of day 20	6.31	6.25	6.29	6.20	0.05	0.58	0.86	0.9
Lactose, %	Colostrum	4.90	4.59	5.17	4.65	0.09	0.82	0.74	0.1
	Milk of day 7	6.61	6.46	6.60	6.17	0.09	0.088	0.16	0.20
	Milk of day 20	6.73	6.68	6.54	6.85	0.07	0.71	0.40	0.45
Total solids content, %	Colostrum	30.01	31.19	29.30	30.91	0.47	0.83	0.92	0.49
	Milk of day 7	27.53	29.01	28.19	28.78	0.29	0.24	0.40	0.32
	Milk of day 20	27.48	27.38	27.87	27.85	0.15	0.27	0.54	0.6
Moisture, %	Colostrum	69.99	68.81	70.70	69.09	0.47	0.83	0.92	0.49
	Milk of day 7	72.47	70.99	71.81	71.22	0.29	0.24	0.40	0.32
	Milk of day 20	72.52	72.62	72.13	72.15	0.15	0.27	0.54	0.6
Solid not fat, %	Colostrum	20.75	21.41	20.09	22.11	0.44	0.42	0.59	0.38
	Milk of day 7	16.46	17.08	16.59	16.67	0.09	0.87	0.50	0.16
	Milk of day 20	16.50	16.37	16.31	16.48	0.06	0.84	0.32	0.61

^a^The number of samples of sows in each group was more than 9 but not equal; SEM, standard error of the mean

### Methionine metabolites in the plasma of sows

On the day of farrowing, no significant differences were found for the concentrations of methionine, homocysteine, cysteine, taurine and glutathione in the plasma of sows in the different groups (Table 5). The levels of methionine increased gradually with an increased Met:Lys ratio in the lactation diet. Moreover, the levels of methionine in the plasma of sows fed with the 0.57-ratio diet were significantly higher than those in the other three groups (*P* < 0.01). The levels of methionine in the plasma of sows fed 0.37- or 0.47-ratio diets were significantly higher than those in the 0.27-ratio group (*P* < 0.01). Homocysteine levels in plasma decreased with the progression of lactation. On the 7^th^ day of lactating and weaning day, the plasma homocysteine level in the 0.57-ratio group was significantly higher than that in the 0.27-ratio group (Table. 4). Moreover, on weaning day, the levels of glutathione and taurine in the plasma increased with the increased Met:Lys ratio (*P* < 0.01). However, the concentration of cystine decreased gradually with an increased dietary Met:Lys ratio on day 7 of lactation and weaning day.

**Table 5:** Effect of dietary Met:Lys ratio in the lactation diet on the levels of methionine metabolites in the blood of sows

Items	Met:Lys				SEM	*P*-value		
	0.27	0.37	0.47	0.57		Linear	Quadratic	Feed
No. of sows^1^								
At parturition								
Methionine, μmol/L^3^	26.88	27.97	34.11	34.37	1.92	0.11	0.27	0.43
Homocysteine, μmol/L^3^	26.78	27.40	26.82	25.47	0.76	0.52	0.67	0.85
Cystine, μmol/L	10.70	9.51	10.47	7.92	0.60	0.36	0.18	0.39
Taurine, μmol/L^3^	52.2	46.1	55.7	50.0	2.0	0.89	0.87	0.85
Glutathione, μmol/L	74.58	76.77	77.77	79.34	1.52	0.75	0.27	0.55
At day 7								
Methionine, μmol/L^2,3^	21.96^C^	39.57^B^	49.63^B^	63.84^A^	2.75	0.00	0.00	0.00
Homocysteine, μmol/L^2,3^	15.88^B^	15.40^B^	17.70^AB^	19.76^A^	0.47	0.00	0.00	0.00
Cystine, μmol/L^2^	6.58^ab^	7.16^a^	5.78^b^	5.49^b^	0.22	0.01	0.03	0.02
Taurine, μmol/L^3^	43.3	61.1	72.7	79.9	5.2	0.01	0.03	0.07
Glutathione, μmol/L	55.14	53.55	55.34	54.76	0.73	0.92	0.95	0.84
At weaning								
Methionine, μmol/L^2,3^	12.21^D^	26.48^C^	36.55^B^	52.99^A^	2.40	0.00	0.00	0.00
Homocysteine, μmol/L^2,3^	13.88^B^	14.55^B^	15.40^B^	18.96^A^	0.48	0.00	0.00	0.00
Cystine, μmol/L^2^	3.90^ab^	4.11^a^	3.40^b^	3.34^b^	0.11	0.01	0.04	0.02
Taurine, μmol/L^2,3^	23.3^B^	36.6^B^	30.4^B^	64.7^A^	3.8	0.00	0.00	0.00
Glutathione, μmol/L^2^	59.31^B^	59.04^B^	58.04^B^	64.97^A^	0.77	0.02	0.00	0.00

^1^The number of samples of sows in each group was more than 9 but not equal; SEM, standard error of the mean

^2^Means in the same row with different letters differ significantly; the different lowercase letters represent *P* < 0.05, and the different capital letters represent *P* < 0.01

^3^There was a treatment × sampling day interaction for plasma methionine (*P* < 0.001), homocysteine (*P* < 0.05) and taurine (*P* < 0.05) concentrations

### Methionine metabolites in the colostrum and milk of sows

The effects of the dietary Met:Lys ratio on methionine metabolites in milk are shown in Table 6. The concentrations of methionine in the colostrum and milk were not influenced by the dietary Met:Lys ratio (*P* > 0.05). However, on day 20 of lactation, the levels of homocysteine in milk increased in those fed an increased dietary Met:Lys ratio (*P* < 0.01). On day 20 of lactation, the cysteine levels in milk from sows in the 0.37- or 0.47-ratio groups were higher than those in the 0.27-ratio group (*P* = 0.050). Furthermore, on day 7 and day 20 of lactation, the milk concentrations of taurine and glutathione, respectively, in the 0.57-ratio group were higher than those in the 0.27-ratio group (*P* < 0.05).

**Table 6:** Effect of dietary Met:Lys ratio in the lactation diet on the levels of methionine metabolites in the colostrum and milk of sows

Items	Met:Lys				SEM	*P*-value		
	0.27	0.37	0.47	0.57		Linear	Quadratic	Feed
No. of sows^1^								
Colostrum								
Methionine, μmol/L	3.28	4.69	3.48	3.20	0.42	0.71	0.64	0.62
Homocysteine, μmol/L	25.66	25.18	38.99	35.29	2.93	0.10	0.26	0.46
Cystine, μmol/L	0.68	0.76	0.86	0.40	0.09	0.35	0.17	0.26
Taurine, μmol/L^3^	575.7	501.7	552.8	552.4	34.7	0.95	0.90	0.92
Glutathione, μmol/L	53.06	52.72	51.88	50.23	0.58	0.07	0.16	0.31
Milk at day 7								
Methionine, μmol/L	14.18	16.30	16.13	18.56	0.74	0.05	0.15	0.23
Homocysteine, μmol/L	30.39	38.19	30.62	32.99	2.33	0.96	0.90	0.66
Cystine, μmol/L	2.11	2.39	2.62	2.24	0.14	0.67	0.77	0.64
Taurine, μmol/L^3^	780.4	944.7	851.9	790.4	24.7	0.70	0.08	0.07
Glutathione, μmol/L	45.63	47.36	47.41	50.16	0.58	0.01	0.03	0.04
Milk at day 20								
Methionine, μmol/L	18.41	18.48	16.77	16.49	0.93	0.38	0.68	0.82
Homocysteine, μmol/L^2^	9.58^B^	20.46^A^	25.01^A^	29.38^A^	2.08	0.00	0.00	0.00
Cystine, μmol/L	2.01	3.39	3.40	2.82	0.21	0.20	0.02	0.05
Taurine, μmol/L^2,3^	801.0^b^	965.3^a^	927.9^ab^	1068.4^a^	29.4	0.01	0.02	0.02
Glutathione, μmol/L	50.19	50.27	52.29	51.69	0.73	0.32	0.59	0.68

^1^The number of samples of sows in each group was more than 9 but not equal; SEM, standard error of the mean

^2^Means in the same row with different letters differ significantly; the different lowercase letters represent *P* < 0.05, and the different capital letters represent *P* < 0.01

^3^There was a treatment × sampling day interaction trend for the level of taurine in milk (*P* = 0.074)

### Oxidative stress parameters in sows

Table 7 shows that the levels of GSH-Px, T-AOC, 8-OHdG and TBARS were significantly affected by dietary treatments (*P* < 0.05), but no differences were observed in the contents of ROS among treatments during lactation (*P* > 0.05). On day 7 of lactation and weaning day, the levels of GSH-Px in the plasma of sows in the 0.37- and 0.47-ratio groups were higher than those in the 0.27-ratio group (*P* < 0.01). The 0.57-ratio diet improved the GSH-Px level on weaning day compared with the 0.27-ratio diet. For T-AOC, compared with the 0.27-group, the levels were remarkably improved in the 0.37-, 0.47- and 0.57-ratio groups at day 7 of lactation and improved in the 0.37- and 0.47-ratio groups at day 14 (*P* < 0.01). Although the levels of ROS in the plasma of sows at different stages of lactation showed no difference (*P* > 0.05), the levels of 8-OHdG were significantly lower in the 0.27- and 0.37-ratio groups than those in the 0.47- and 0.57-ratio groups on day 7 of lactation (*P* < 0.05), while the lowest level of 8-OHdG was observed in the 0.37-ratio group on the day of weaning. Moreover, the contents of TBARS were increased in sows fed the 0.57-ratio diet compared with those fed a lower Met:Lys ratio diet (*P* < 0.05 at day 7, *P* = 0.06 at weaning day).

**Table 7:** Effect of dietary Met:Lys ratio in the lactation diet on oxidative stress parameters of sows

Items	Met:Lys				SEM	*P*-value		
	0.27	0.37	0.47	0.57		Linear	Quadratic	Feed
No. of sows^1^								
At parturition								
GSH-Px, U/mL^3^	843.73	800.09	768.65	811.04	16.33	0.38	0.28	0.44
T-AOC, U/mL^3^	0.77	0.59	0.98	0.95	0.06	0.07	0.44	0.08
ROS, U/mL	36.49	30.83	41.48	41.65	2.27	0.44	0.62	0.30
8-OHdG, ng/mL	37.44	33.07	37.72	33.26	1.83	0.66	0.91	0.70
TBARS, nmol/mL	3.08	3.1	2.81	2.71	0.16	0.35	0.60	0.79
At day 7								
GSH-Px, U/mL^2,3^	672.88^B^	811.65^A^	763.09^A^	621.40^B^	16.91	0.19	0.00	0.00
T-AOC, U/mL^2,3^	0.30^C^	0.58^B^	1.00^A^	0.50^BC^	0.06	0.04	0.00	0.00
ROS, U/mL	34.66	32.54	39.11	38.22	1.87	0.21	0.41	0.12
8-OHdG, ng/mL^2^	50.05^a^	43.08^ab^	38.47^b^	38.78^b^	1.60	0.01	0.01	0.03
TBARS, nmol/mL^2^	3.82^b^	3.78^b^	4.51^ab^	4.73^a^	0.15	0.01	0.02	0.04
At weaning								
GSH-Px, U/mL^2,3^	632.84^C^	895.69^A^	773.88^B^	799.85^B^	17.92	0.03	0.00	0.00
T-AOC, U/mL^2,3^	0.36^B^	0.62^B^	1.27^A^	1.36^A^	0.10	0.00	0.00	0.00
ROS, U/mL	46.20	37.92	39.79	40.64	1.79	0.64	0.54	0.45
8-OHdG, ng/mL^2^	50.56^a^	40.58^b^	49.15^a^	50.42^a^	1.37	0.58	0.31	0.02
TBARS, nmol/mL	2.47	2.24	2.67	3.65	0.19	0.03	0.02	0.17

^1^The number of samples of sows in each group was more than 9 but not equal; SEM, standard error of the mean;

^2^Means in the same row with different letters differ significantly; the different lowercase letters represent *P* < 0.05, and the different capital letters represent *P* < 0.01;

^3^There was a treatment × sampling day interaction for plasma GSH-Px and T-AOC concentrations (*P* < 0.01), and the levels of GSH-Px and T-AOC in plasma at parturition and weaning were significantly higher than those at day 7 (*P* < 0.001);

*GSH-Px* glutathione peroxidase, *T-AOC* total antioxidant capacity, *ROS* reactive oxygen species; *8-OHdG* 8-hydroxy-deoxyguanosine; *TBARS* thiobarbituric acid reactive substances

## Discussion

Sow feed intake during lactation has a significant impact on reproductive performance [14]. Many studies have shown that increased feed intake decreases body weight loss as well as increases the backfat depth [15, 16]. In the current study, we showed that increasing the dietary Met:Lys ratios in the lactation diet did not impact the WEI, weight loss or backfat thickness loss of sows during lactation, which is consistent with previous studies [17].

It is well known that lactating sows need abundant nutrients to maintain lactation and need essential nutritional reserves for subsequent reproduction. Amino acids are essential for the normal development of mammary glands and milk synthesis in lactating sows [18]. A previous study showed that the methionine level recommended by the NRC (2012) may be insufficient for supporting optimal milk yield and milk protein synthesis in lactating sows [6]. However, our research results showed that increased dietary Met:Lys ratios did not affect the total milk yield or the milk protein content. Moreover, the fat, lactose, nonfat solids and total solids in the colostrum or milk were also not influenced by the dietary Met:Lys ratio.

Since the milk yield and nutrient content in milk remained unchanged, the litter weight was not affected by the dietary treatments during the lactation period. However, piglet weights weaned from sows fed a 0.47-ratio diet were significantly higher than those weaned from sows fed a 0.27-ratio diet (7.55 kg vs. 6.85 kg, Table 3). The improved piglet weight might be caused by the lower weekly litter size of sows in the 0.47-group.

Methionine, an essential sulfur-containing amino acid, not only plays an important role in protein synthesis but also influences the antioxidative system due to its metabolite glutathione, which is generated via sulfur metabolism. In the current study, the results showed that increasing the dietary Met:Lys ratio in the lactation diet improved the content of methionine in plasma and facilitated sulfur metabolism to form glutathione during the entire lactation period (Table 5). However, oxidative status biomarkers, ROS, in the plasma of sows were unaffected by the dietary Met:Lys ratio. Notably, another metabolite, homocysteine, is readily oxidized in the body to cause oxidative stress [19]. In the present study, the concentrations of homocysteine in plasma were increased with an increased Met:Lys ratio. This is in accordance with research that reported that an increase in the methionine level in the diet would enhance sulfur metabolism [20]. The increased homocysteine level might abolish the antioxidative effect of glutathione.

Moreover, in the present study, we showed that the homocysteine content increased dramatically in the 0.37-, 0.47- and 0.57-ratio groups on day 20 of lactation (*P* < 0.01). We showed that the homocysteine levels in milk were greater than those in the plasma during the lactation period, which could be attributed to the lower activity of enzymes involved in homocysteine metabolism. However, there is no available information on the expression or activity of these enzymes in porcine mammary glands. Growing evidence has demonstrated that the level of homocysteine is positively correlated with the content of ADMA (asymmetrical dimethyl arginine) [21], which is an endogenous inhibitor of inducible nitric oxide synthase to form nitric oxide (NO) [22, 23]. Since the important role of NO in regulation of blood flow, it has been proposed that increased homocysteine decreased regional blood flow [24]. However, in the current study, the increased homocysteine did not decreased total milk yield or the milk protein content, suggesting that the blood flow might not affect by the changed homocysteine levels. Interestingly, it has been shown that GSH enhanced production of NO in rodents and humans [25]. Hence, we speculate that No production might remain balance when both GSH and homocysteine levels was increased in the current study, but further testing is necessary for confirmation.

**Fig. 2:**
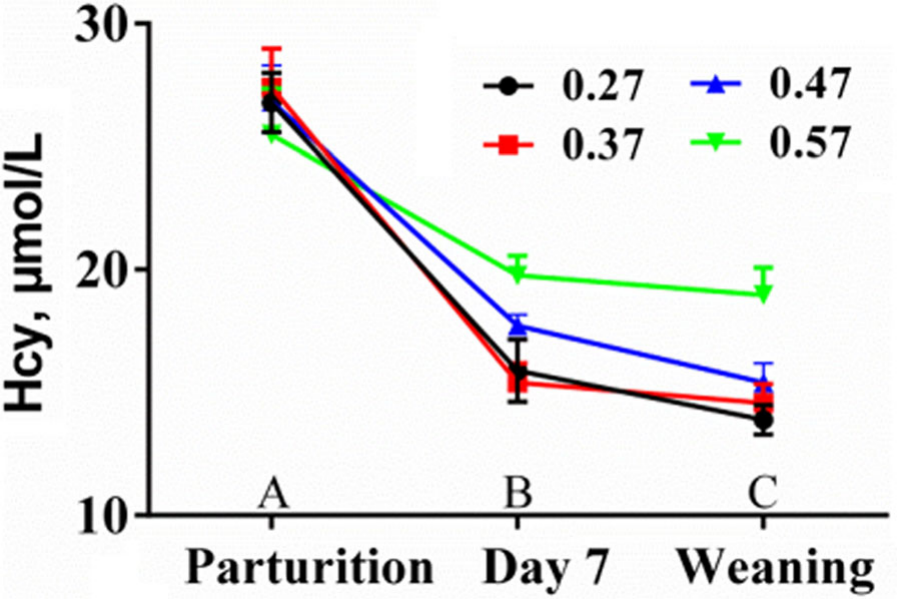
The trend change in homocysteine (Hcy) in plasma along with the lactation process. Blood samples were collected from the ear vein of sows at the morning feeding and 2 h later. Data are presented as the means±SEM, *n* ≥ 11/lactation diet. ^A-C^Means in the same row with different letters differ significantly, *P* < 0.01

Surprisingly, the concentrations of homocysteine in the colostrum and milk on day 7 were high, indicating large-scale homocysteine accumulation in the mammary gland cells of perinatal sows. Furthermore, the levels of homocysteine in the plasma of sows at parturition were high and decreased gradually as lactation progressed (Fig. 2); interestingly, this trend is almost the same as that of ROS during lactation in sows [8]. Thus, we speculate that high levels of homocysteine in the plasma of perinatal sows may be an important potential factor for high oxidative stress during the perinatal period, as homocysteine is easily oxidized in the body [19]. Therefore, these results provide a new direction for further research on the mechanism of sow systemic oxidative stress and the optimization of nutrition regulation strategies for sows.

## Conclusion

The current study indicated that increasing the dietary Met:Lys ratio (0.37~0.57) in the lactation diet had no significant effect on the overall performance of sows or the colostrum and milk composition, but it increased piglet mean BW and piglet ADG during the first week of lactation. Increasing dietary methionine levels increased levels of homocysteine GSH and GSH-Px in the plasma of sows, but had no significant effect on antioxidant status in sows.

## Data Availability

The datasets analyzed are not publicly available due to ownership by the funding partners but are available from the corresponding author upon reasonable request.

## Abbreviations

8-OHdG: 8-hydroxy-2′-deoxyguanosine
ADG: Average daily gain
ADMA: Asymmetrical dimethyl arginine
BW: Body weight
GSH: Glutathione
GSH-Px: Glutathione peroxidase
Hyc: Homocysteine
IAA: Indispensable amino acid
Lys: Lysine
Met: Methionine
NO: Nitric oxide
NOS: Nitric oxide synthase
NRC: National Research Council
PUN: Plasma urea nitrogen
ROS: Reactive oxygen species
SAA: Sulfur amino acid
SID: Standardized ileal digestible basis
T-AOC: Total antioxigenic capacity
TBARS: Thiobarbituric acid reactive substances
WEI: Weaning to estrous interval

## References

[1] Strathe AV, Bruun TS, Zerrahn JE, Tauson AH, Hansen CF (2016). The effect of increasing the dietary valine-to-lysine ratio on sow metabolism, milk production, and litter growth. J Anim Sci.

[2] Shoveller AK, Stoll B, Ball RO, Burrin DG (2005). Nutritional and functional importance of intestinal sulfur amino acid metabolism. J Nutr.

[3] Fang Z, Huang F, Luo J, Wei H, Ma L, Jiang S (2010). Effects of dl-2-hydroxy-4-methylthiobutyrate on the first-pass intestinal metabolism of dietary methionine and its extra-intestinal availability. Br J Nutr.

[4] Bauchart-Thevret C, Stoll B, Chang X, Cui L, Burrin D (2008). Sulfur amino acids are necessary for normal intestinal mucosal growth in neonatal piglets. Federation of American Societies for Experimental Biology.

[5] Bauchart-Thevret Caroline, Stoll Barbara, Chacko Shaji, Burrin Douglas G. (2009). Sulfur amino acid deficiency upregulates intestinal methionine cycle activity and suppresses epithelial growth in neonatal pigs. American Journal of Physiology-Endocrinology and Metabolism.

[6] Guan X, Bequette BJ, Ku PK, Tempelman RJ, Trottier NL (2004). The amino acid need for milk synthesis is defined by the maximal uptake of plasma amino acids by porcine mammary glands. J Nutr.

[7] Berchieri-Ronchi C, Kim S, Zhao Y, Correa C, Yeum KJ, Ferreira A (2011). Oxidative stress status of highly prolific sows during gestation and lactation. Animal..

[8] Tan Chengquan, Wei Hongkui, Sun Haiqing, Ao Jiangtao, Long Guang, Jiang Siwen, Peng Jian (2015). Effects of Dietary Supplementation of Oregano Essential Oil to Sows on Oxidative Stress Status, Lactation Feed Intake of Sows, and Piglet Performance. BioMed Research International.

[9] Stanger O, Weger M (2003). Interactions of homocysteine, nitric oxide, folate and radicals in the progressively damaged endothelium. Clin Chem Lab Med.

[10] Misra HP (1974). Generation of superoxide free radical during the autoxidation of thiols. J Biol Chem.

[11] Upchurch GR, Welch GN, Fabian AJ, Freedman JE, Johnson JL, Keaney JF (1997). Homocyst (e) ine decreases bioavailable nitric oxide by a mechanism involving glutathione peroxidase. J Biol Chem.

[12] Fang ZF, Luo J, Qi ZL, Huang FR, Zhao SJ, Liu MY (2009). Effects of 2-hydroxy-4-methylthiobutyrate on portal plasma flow and net portal appearance of amino acids in piglets. Amino Acids.

[13] Wang SP, Yin YL, Qian Y, Li LL, Li FN, Tan BE (2011). Effects of folic acid on the performance of suckling piglets and sows during lactation. J Sci Food Agric.

[14] Lucia T, Dial GD, Marsh WE (2000). Lifetime reproductive performance in female pigs having distinct reasons for removal. Livest Prod Sci.

[15] Eissen J, Apeldoorn E, Kanis E, Verstegen M, De Greef K (2003). The importance of a high feed intake during lactation of primiparous sows nursing large litters. J Anim Sci.

[16] Peng J, Somes S, Rozeboom D (2007). Effect of system of feeding and watering on performance of lactating sows. J Anim Sci.

[17] Schneider JD, Nelssen JL, Tokach MD, et al. Determining the total sulfur amino acid to lysine requirement of the lactating sow[J]. Kansas Agricultural Experiment Station Research Reports. 2006;(10):47-51.

[18] Kim SW, Mateo RD, Yin YL, Wu G (2007). Functional amino acids and fatty acids for enhancing production performance of sows and piglets. Asian Australas J Anim Sci.

[19] Refsum H, Ueland PM (1990). Clinical significance of pharmacological modulation of homocysteine metabolism. Trends Pharmacol Sci.

[20] Stipanuk MH (2004). Sulfur amino acid metabolism: pathways for production and removal of homocysteine and cysteine. Annu Rev Nutr.

[21] Atamer A, Ecder SA, Atamer Y, Kocyigit Y, Yigit NB, Ecder T. The Effects of Asymmetric Dimethylarginine (ADMA), Nitric Oxide (NO) and Homocysteine (Hcy) on Progression of Mild Chronic Kidney Disease (CKD): Relationship Between Clinical and Biochemical Parameters. Chronic Kidney Dis. 2012.

[22] Boger RH, Lentz SR, Bode-Boger SM, Knapp HR, Haynes WG (2001). Elevation of asymmetrical dimethylarginine may mediate endothelial dysfunction during experimental hyperhomocyst (e) inaemia in humans. Clin Sci.

[23] Zhang X, Li H, Liu G, Wan H, Mercier Y, Wu C (2015). Differences in plasma metabolomics between sows fed dl-methionine and its hydroxy analogue reveal a strong association of milk composition and neonatal growth with maternal methionine nutrition. Br J Nutr.

[24] Toda N, Okamura T (2016). Hyperhomocysteinemia impairs regional blood flow: involvements of endothelial and neuronal nitric oxide. Pflugers Arch - Eur J Physiol.

[25] McKinley-Barnard S, Andre T, Morita M, Willoughby DS (2015). Combined L-citrulline and glutathione supplementation increases the concentration of markers indicative of nitric oxide synthesis. J Int Soc Sports Nutr.

